# Development and implementation of a high-fidelity simulation training course for medical and nursing collaboration based on the Fink integrated course design model

**DOI:** 10.3389/fmed.2024.1286582

**Published:** 2024-03-05

**Authors:** Meng-Han Jiang, Li-Wen Dou, Bo Dong, Man Zhang, Yue-Ping Li, Cui-Xia Lin

**Affiliations:** ^1^School of Health, Shandong University of Traditional Chinese Medicine, Jinan, Shandong, China; ^2^School of Political Science and Public Administration, Wuhan University, Wuhan, Hubei, China; ^3^Peking University First Hospital, Beijing, China; ^4^School of Nursing, Shandong Modern University, Jinan, Shandong, China

**Keywords:** Fink integrated curriculum design model, collaborative healthcare education, high fidelity simulation, curriculum design, collaborative healthcare attitudes, medical and nursing collaboration, critical thinking

## Abstract

**Aim:**

The purpose of this study is to examine the design and implementation of a high-fidelity simulation training course for medical and nursing collaboration, based on the Fink integrated course design model. Additionally, the study aims to validate the teaching effectiveness of the course.

**Background:**

Previous empirical studies have highlighted the effectiveness of collaborative healthcare education in institutional teaching and hospital training. However, the development of healthcare collaborative education in China has been slow to develop in China. In recent years, Chinese nursing educators and researchers have shown interest in utilizing high-fidelity simulators for healthcare collaborative education. These simulators help address the limitations of traditional nursing teaching and healthcare separation simulation. Nevertheless, a standardized simulation interprofessional education curriculum is still lacking. Therefore, nursing educators need to develop a standardized high-fidelity simulation training curriculum for healthcare collaboration, guided by established science curriculum development theories.

**Methods:**

A high-fidelity simulation training course on healthcare collaboration was designed based on the Fink integrated curriculum design model. The course was taught to 14 nursing students and 8 clinical medicine students from March to July 2022. To comprehensively evaluate the effectiveness of the healthcare collaboration high-fidelity simulation training course, several assessment tools were used. These included course grades, satisfaction and self-confidence scales, simulation teaching practice scales, healthcare collaboration attitude scales, critical thinking skills scales, and semi-structured interviews.

**Results:**

After the course was implemented, students demonstrated high overall scores (79.19 ± 5.12) and reported high satisfaction ratings (4.44 ± 0.37). They also exhibited increased self-confidence (4.16 ± 0.33). Additionally, students evaluated all four dimensions of the course teaching practice scale positively. Furthermore, the study demonstrated significant improvements in various aspects, such as attitudes toward medical and nursing collaboration (*t* = −7.135, *P* < 0.01), shared education and teamwork (*t* = −3.247, *P* = 0.002), job autonomy for nurses (*t* = −1.782, *P* = 0.000), and reduced physician dominance (*t* = −6.768, *P* = 0.000). The critical thinking skills of the students showed significant improvement, with higher scores in truth-seeking (*t* = −3.052, *P* = 0.004), analytical ability (*t* = −2.561, *P* = 0.014), systematic ability (*t* = −3.491, *P* = 0.001), self-confidence in critical thinking (*t* = −4.024, *P* = 0.000), and curiosity (*t* = −5.318, *P* = 0.000) compared to their scores before the course (all *P* < 0.05). The interviews showed that the course’s student-centered approach enabled active learning. Students suggested enhancing teaching cases and allocating more time for reflection and summarization.

**Conclusion:**

The study successfully designed a high-fidelity simulation training course for healthcare collaboration by utilizing the Fink integrated curriculum design model. The findings provide valuable insights for the development of standardized curricula and healthcare collaboration education in China. Moreover, the course adheres to best practice principles, fostering improved attitudes toward healthcare collaboration and enhancing students’ healthcare collaboration and clinical thinking skills.

## 1 Introduction

Modern medical personnel training models emphasize the need to strengthen teamwork and promote interprofessional education (1). Interprofessional education, which was first proposed in the United Kingdom during the 1960s, has gained continuous support and development by organizations such as the World Health Organization (WHO) (2). Currently, interprofessional education involves extensive collaboration between institutions and regions (3, 4).

Collaborative healthcare education is a type of interprofessional education where nursing and clinical medicine students learn from each other. The goal is to improve patient health outcomes by strengthening collaboration between healthcare professionals (5). Studies conducted overseas have confirmed the positive effects of collaborative healthcare education on improving students’ skills and non-skills. For example, Oxelmark et al. (6) researchers used five clinically common scenarios of interprofessional collaboration scenarios, such as post-operative hemorrhage and allergic reactions, to improve the ability of clinical medical students and nursing students to collaborate during emergencies. Similarly, in a study conducted by Jakobsen et al. (7), nursing students, anesthesia nurses, and clinical medical students underwent interprofessional training. The results showed that the students were able to adapt to their team roles better and enhance their non-technical skills. Lau et al. (8) conducted a 2-day interprofessional advanced cardiovascular life support training for nursing and clinical medicine students. The results showed that the training improved students’ team performance, communication skills, and ability to work effectively in acute and critical care situations. In contrast, collaborative healthcare education in China has only been reported in the early 21st century, with research still in its early stages (9).

Scenario-based simulation can provide a safe healthcare environment for collaborative healthcare education and enable students to improve their practical skills in real-life situations. In recent years, the development of situational simulation teaching has garnered attention from nursing educators and researchers in China, particularly in the realm of medical-nursing collaborative education based on high-fidelity simulators. Wang et al. (10) investigated the effectiveness of high-fidelity simulation in teaching operating room nursing collaboration. Other researchers have also applied this method in nursing planning and implementation (11) and emergency nursing courses (12). The results demonstrate that this teaching method can enhance students’ interest in learning and improve their teamwork skills. Currently, China’s high-fidelity simulation teaching of healthcare collaboration is still in the developmental stage. Most researchers design the teaching content based on the actual needs and available resources of their institutions. The teaching is mostly carried out by focusing on one or more trainings in a nursing specialty course (13–15). However, this approach may lack scientific rigor in the teaching process and make it difficult to compare teaching effects horizontally.

Since curriculum development is the initial step in implementing curriculum teaching, and its quality directly affects the curriculum’s implementation, nursing educators must standardize the development of a high-fidelity simulation training course for healthcare collaboration under the guidance of scientific curriculum development theories. Studies have shown that educators, both domestic and international, have adopted curriculum development theories to guide the process. For instance, some have used the flexible learning model to design a health assessment course (16), while others have developed their own model based on competency-based education theory (17).

However, one integrated curriculum design model (Below is referred to as the “Fink model”) that has emphasized the creation of meaningful learning experiences as a key aspect of quality education was developed by Fink (18). The model is holistic, comprehensive, and practical, focusing on both theoretical exploration and conceptual analysis, as well as concrete implementation to improve teaching effectiveness (19).

The Fink model has been successful in a variety of fields, including basic dental anatomy courses (20), health policy courses (21), and narrative nursing courses (22). In this study, the Fink model served as the theoretical basis for developing a high-fidelity simulation training course for healthcare collaboration, offering several benefits: (1) This tool assists educators in analyzing the course needs to clarify the course’s nature and curriculum significance objectively. (2) Instead of traditional goal-setting, this tool employs meaningful learning objectives. (3) The course evaluation elements align with the formative and summative evaluation advocated by the simulation teaching evaluation method. (4) Analyzing whether the course elements can support each other to ensure the course’s systematic nature; and (5) Predicting potential problems that may arise during the course implementation stage to ensure its feasibility.

Based on the need to improve curriculum development for collaborative education, a SimMan3G (SimMan3G is actually a high-fidelity mannequin from Laerdal) has been developed as an integrated simulator-based healthcare cooperation training curriculum using Fink’s design model. This study aims to explore the development, implementation, and evaluation of the SimMan3G in teaching nursing and clinical medicine students. The findings will provide valuable insights for standardizing the development of healthcare collaboration curriculum, cultivating students’ awareness of healthcare collaboration, and enhancing their healthcare collaboration skills.

## 2 Materials and methods

This study is divided into two parts: curriculum development and curriculum implementation. Firstly, we explored the process of developing a SimMan3G-based collaborative healthcare training course using the Fink model. Secondly, we implemented the curriculum with students from two specialties, clinical medicine and nursing, as research subjects and verified its teaching effectiveness.

### 2.1 Course development

#### 2.1.1 Theoretical basis

The Fink integrated curriculum design model consists of three phases (18), outlined in Table 1. Each phase includes specific operational steps to guide educators through the curriculum development process. The initial stage is particularly important and serves as the foundation for designing a course. To guide the development of a SimMan3G-based healthcare collaboration training course using the Fink model, instructional designers should first analyze contextual factors to understand the current status of healthcare collaboration in the nursing field in China. Then, they should determine meaningful learning objectives for the course and select appropriate feedback assessment procedures and effective teaching activities based on the course objectives. The intermediate phase aims to integrate foundational elements into a dynamic and coherent whole. The final phase aims to enhance the curriculum design.

**TABLE 1:** Fink integrated curriculum design model content.

Stage	Main steps
Initial phase: determining the foundational elements of the course	Clarify contextual factors
	Define learning objectives
	Develop appropriate feedback and evaluation systems
	Designing teaching activities
	Integration of the identified basic components of the curriculum
Intermediate phase: integration of essential factors into the whole	Designing course structure
	Choosing effective teaching strategies
	Designing an overall learning activity plan
Final phase: completion of other important tasks	Establishment of a scoring system
	Identify issues that may arise
	Completing a course outline
	Planned assessment of curriculum and instruction

#### 2.1.2 Course construction

This study presents the development of a high-fidelity training course for medical and nursing collaboration in three stages: initial, intermediate, and final. The Fink model was used as a basis for this construction. The analysis of each stage is presented below:

(1) Initial stage

The contextual factors of the course include six specific aspects. (1) External Expectations: The aim of this course is to address the issue of neglecting healthcare collaboration in nursing practical training courses and promote teaching reform in the nursing profession. (2) Specific Context: This course was proposed in the context of the new medical science background (23) and the specific context of China’s relatively lagging development of education on healthcare collaboration. (3) Course Nature: The course is an interprofessional elective course on medical situational simulation, which emphasizes the cultivation of teamwork attitudes and abilities among nursing and clinical medical students. (4) Student Characteristics: The students are senior-level and possess professional knowledge and basic operational skills. They can analyze cases based on their own understanding. (5) Teacher characteristics: the teachers all have the title of associate senior and above and rich experience in simulation teaching, and they can instruct the students how to use SimMan3G for training. (6) Teaching special challenges: the SimMan3G integrated simulation system can’t meet the actual needs of the teaching content of the course. As a result, the School of Nursing, the School of Clinical Medicine, and the teaching hospital collaborated in the preliminary stage to jointly prepare eight teaching cases based on certain case preparation principles and processes (24).

Fink emphasizes the importance of meaningful learning in teaching practices and has created six taxonomies to achieve this: basics, applications, synthesis, humanities, caring, and learning to learn. When determining the course’s total learning objectives based on this taxonomy, teachers should focus not only on students’ understanding of the basics but also on developing their application skills and other levels (25). The study developed the courses’ learning objectives, which are listed in Table 2.

**TABLE 2:** Total learning objectives of the medical-nursing collaborative high-fidelity simulation training course.

Dimensionality	Course objectives
Basic knowledge	Master the basic theoretical knowledge of case-related diseases and diagnostic and treatment (nursing) measures, familiar with the assessment, diagnosis, and treatment (nursing) plan development
Applications	Ability to perform specialized skills in related diseases and to work effectively with team members in the development of diseases
Synthesis	Ability to think about the connections between the 2 disciplines, the meaning of division of labor and collaboration, and how to apply collaborative thinking and skills in healthcare to future clinical work
Humanities	To be able to recognize the role of the learning process, to improve the attitude of cooperation between healthcare and nursing, and to take the code of professional ethics as the guiding code of conduct, reflecting the humanistic care for patients
Caring	Be curious and motivated by the phenomena, ideas, and learning process of the content being studied
Learning to Learn	Build knowledge through reflection and promote independent learning while strengthening the effect of simulation teaching

The course was evaluated using three methods: prospective assessment, self-assessment, and FIDeLity feedback (Frequent, Immediate, Discriminating based on criteria and standards, Delivered Lovingly or supportively). A questionnaire was used to assess changes in students’ attitudes toward healthcare cooperation and critical thinking skills before and after the course implementation. The instructor conducted summative scoring of group-recorded case videos using a self-designed key competency checklist. The checklist includes 5 areas: team decision-making, communication, situational monitoring, mutual support, and first aid, with 20 points allocated to each area. The checklist was used to develop students’ self-assessment skills. Additionally, the instructor utilized a Context-Content-Course (3C) guided feedback model (26) to encourage students’ analysis and reflection during high-fidelity simulation training sessions. The course included various active learning activities such as independent review of theoretical knowledge and skills related to the case, role-playing, collaborative learning, high-fidelity simulation training, and guided feedback. The course facilitated student learning through three areas: gaining information and perspectives, experiencing, and reflecting.

A review form based on the Fink design was used to examine a high-fidelity simulation training course on healthcare collaboration. The course addresses the learning objectives and selects appropriate feedback and assessment methods and instructional activities. The foundational elements were able to support each other and work together to promote meaningful learning.

(2) Intermediate stage

The course was an elective and consisted of two topics: introduction and case study. The introduction topic was allocated 2 h, while each of the 8 cases was assigned 4 h, resulting in a total of 34 h of instruction. The course employed a “team-based learning” strategy, leveraging the SimMan3G integrated simulator to simulate real clinical situations. Students worked in groups to engage in high-quality applied learning for the cases. The course design consisted of four components: course theme, teaching content, teaching activities, and credit hours, as shown in Table 3.

**TABLE 3:** Overall plan of the medical-nursing collaborative high-fidelity simulation training course.

Sessions	Topics	Teaching content	Teaching activities	Credit hours
1	Introduction	Introduction to the course (objectives, teaching arrangements, evaluation methods), explanation of the application of the simulation system (simulators, scene layout, simulation fidelity, how students observe during the simulation)	Lecture method	2
2	Case study	Acute myocardial infarction	Case studies, Roleplay, Collaborative learning, High-quality simulation training, Guided feedback	4
3		Diabetic ketoacidosis		4
4		Perforated duodenal ulcer		4
5		Thyroid Cancer		4
6		Postpartum bleeding in normal labor		4
7		Amniotic fluid embolization		4
8		Neonatal asphyxia resuscitation		4
9		Pediatric severe pneumonia		4

(3) Final stage

After identifying the course elements in the first two stages, the final stage involves determining the course’s teaching assessment, grading system weighting, and completing the course outline. The course outline comprises eight sections: basic information (including course name, total hours, prerequisite courses, applicable target, and course leader), course objectives, teaching content and class schedule, teaching methods, performance assessment methods, recommended teaching materials, connection and division of labor with other courses, and course introduction.

### 2.2 Course implementation

#### 2.2.1 Study population

In March 2022, a teaching class was formed for the study, consisting of students in the fourth year of a 5-year clinical medicine program and the third year of a 4-year nursing program at a university. The recruitment criteria are as follows: (1) Full-time undergraduate clinical medicine and nursing majors; (2) Completion of basic medical courses, including human anatomy, pathology, and physiology. Clinical medicine students have also completed professional courses such as surgery, internal medicine, obstetrics and gynecology, and pediatrics. Nursing students have completed courses such as surgical nursing, internal medicine nursing, obstetrics and gynecology nursing, and pediatric nursing; (3) No exposure to interprofessional-related content in daily practical training; (4) Experience in simulation learning; (5) Availability to participate in the course; and (6) Understanding of the purpose and significance of the course. Due to time constraints and limited manpower, 22 students were recruited for the initial course development. The participants included 14 nursing students and 8 clinical medicine students, with ages ranging from 20 to 23 years old (mean age of 20.73 ± 0.94 years). The group consisted of 2 male and 20 female participants. In order to further verify the reliability of the data, we have done a power analysis, which shows that the data has good reliability.

#### 2.2.2 Study design

The course implementation is divided into 2 parts: pre-teaching preparation and teaching implementation. Pre-teaching preparation involves preparing the teachers, students, and learning environment. Teaching implementation follows the steps of scenario introduction, high-fidelity simulation training, and review. Take “acute myocardial infarction” for example, the details are described as follows:

(1) Pre-teaching preparation

Each case is taught by a team of instructors consisting of a nursing faculty member, a clinical medicine faculty member, a laboratory faculty member, and a teaching assistant. The instructors conduct an in-depth analysis of the case and prepare a lesson plan in advance. The lesson plan contains a schedule, training objectives, prerequisite knowledge for students, case overview, pre-course preparation (including scene setting, simulators, teaching aids, role division, consultation/nursing aids, and drugs), case trend chart, development process, and review outline. Furthermore, the case and learning tasks are provided to students beforehand. The laboratory instructor imports the case information into the instructor console for the teaching team to pilot. They work with the teaching assistant to provide the necessary equipment and items for the class according to the lesson plan. Before class, students form their own medical and nursing cooperative teams, determine their roles, familiarize themselves with the script, and review the relevant theoretical knowledge and operational skills.

(2) Teaching implementation

**FIGURE 1:**
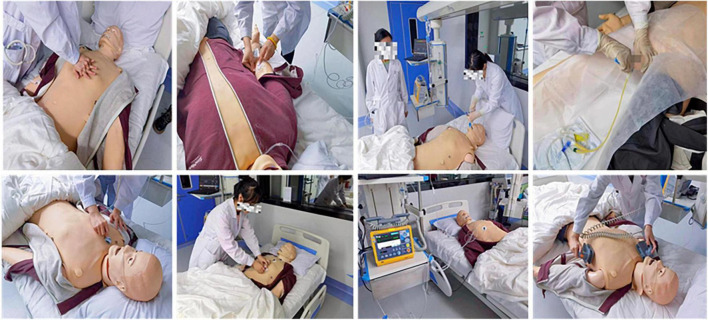
Students undergoing high-fidelity simulation training.

In the introduction scenario link, the teacher presents the students with a high-fidelity simulation training case of acute myocardial infarction healthcare collaboration, as shown in Table 4. The teacher addresses any questions the students may have encountered during their independent study before the class, confirms the role division of students, analyzes the simulation tasks with them, explains the presentation requirements, and encourages students to be fully prepared for the training. During the high-fidelity training session, the teacher initiates the program, and students assume their roles based on the disease progression and tasks in each scenario. This commences the high-fidelity training for medical and nursing collaboration, as shown in Figure 1. One group performs the simulation training while the other groups observe and record through live video in the observation room. During the review session, the teacher and students review the high-fidelity simulation training process together using video replay. The review session consists of two phases: (1) Introduction phase, during which the teacher explains the purpose and steps of the session to the students, and (2) Situational phase. The teacher prompts students to provide feedback on the performance of their peers during high-fidelity training. This is done by asking simple questions such as “How do you feel about the performance of this group of students just now?” (3) The content stage involves presenting objective facts, encouraging open discussion, and providing the teacher’s perspective from the patient’s point of view. (4) The expansion phase follows. Students are instructed to summarize their learning experiences and consider how they can apply what they have learned to their future clinical practice.

**TABLE 4:** Acute myocardial infarction healthcare cooperation high-fidelity simulation practical training case.

Case title	Acute myocardial infarction
Teaching goal	① Cognitive domain: recognize the etiology of acute myocardial infarction, associated risk factors, and clinical manifestations. ② Action skill domain: medical students need to apply the knowledge they have learned to skillfully implement the receiving process, body check, cardiopulmonary resuscitation, bedside electrocardiogram, defibrillation; nursing students need to skillfully implement indwelling catheterization, intravenous fluids, and collection of blood specimens; and medical and nursing students jointly master the resuscitation process. ③ Emotional domain: students embody humanistic care through good communication with patients and their families; through the implementation of treatment as well as nursing measures for patients, students develop a collaborative attitude toward healthcare.
Case description	Patient, male, 61 years old, chief complaint and history: the patient complained of chest pain that suddenly appeared 1 h ago with no obvious cause, the pain site is mainly in the precordial area, and the pain range is about the size of the palm, the pain is pressure-like pain, accompanied by profuse sweating, palpitation, radiating pain in the back of the shoulder and the pharynx, there is no nausea, vomiting, there is no tightness in the chest, shortness of breath, fatigue, there is no coughing, coughing up sputum, hemoptysis, Self-medication “fast-acting heart pills” after the symptoms did not relieve, and he called 120 and came to our hospital urgently. He underwent cardiopulmonary resuscitation and electrocardiogram showed “acute extensive anterior wall myocardial infarction,” and was transferred to our department for thrombolytic therapy. Past history: 10 years history of hypertension and coronary heart disease. Physical examination: temperature 36.5°C, respiration 21 times/min, pulse 90 times/min, blood pressure 90/59 mmHg, clear, superficial lymph nodes are not palpable enlargement, lips and lips without cyanosis, no jugular veins; symmetry of the thorax, the lungs breath sounds thick, heard full lung wet rales, percussion of the cardiac boundary is not big; listening to the rhythm of the heart is synchronous, the valvular auscultation area did not hear a murmur; the abdominal flat and soft, no compression pain and rebound pain The abdomen was flat and soft, with no pressure or rebound pain. The liver and spleen were not palpable, and there was no edema in the lower limbs. The electrocardiogram showed that the V1-V5 ST segments were elevated about 0.3–0.5 mv.
Scenario setting	Scenario 1: out-of-hospital treatment ① Doctor’s task: 120 telephone reception, instructing family members to perform cardiopulmonary resuscitation, bedside electrocardiogram measurement, decision-making, and completion of medical orders; ② Nurse’s task: oxygen supply, establishment of intravenous access, and administration of medication in accordance with medical advice; ③ Medicine and nursing joint task: communication of the patient’s vital signs, and comforting the patient’s family members. Scenario 2: in-hospital emergency care ① Doctor’s task: explain the patient’s condition, bedside electrocardiogram, cardiopulmonary resuscitation, and defibrillation, to complete the doctor’s orders; ② Anesthesiologist’s task: endotracheal intubation, simple respiratory balloon ventilation; ③ nurse’s task: the preparation of resuscitation supplies, coordination of various departments to do a good job of resuscitation preparations, blood sampling, resuscitation records; ④ healthcare common task: communication of the patient’s vital signs Scenario 3: internal medicine treatment ① Doctor’s task: physical examination, asking the family about the patient’s medical history, decision-making about thrombolytic therapy, judgment of the condition; ② Nurse’s task: blood sampling, thrombolytic operation, changing the patient’s position, oxygenation, indwelling catheterization, resuscitation records; ③ Healthcare co-worker’s task: explaining to the patient’s family about the treatment and recommendation for transferring to a different hospital.

#### 2.2.3 Evaluation methods

**FIGURE 2:**
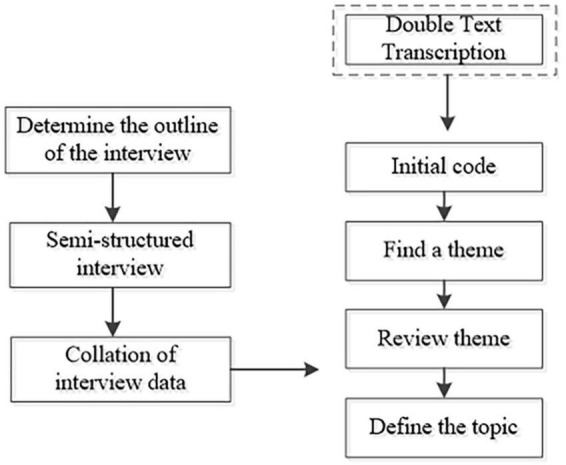
Semi-structured interview research process.

A mixed methods approach is suitable for comprehensively evaluating the SimMan3G collaboration training curriculum. When evaluating the effectiveness of nursing high-fidelity simulation teaching, researchers usually focus on various aspects, including student achievement, course satisfaction, student confidence, teamwork ability, and critical thinking ability (27–29). This study comprehensively assessed the teaching effectiveness of the course based on the following dimensions:

(1) Student Course Grades: The total score is graded out of 100 points. The weight of each assessment component was determined based on the course syllabus and the opinions of the interdisciplinary teaching team. The formative evaluation constitutes 60% of the total student course grade, with 10% for self-evaluation, 20% for peer evaluation, and 30% for teacher evaluation. The remaining 40% is allocated to teacher evaluation of the group recording video.
(2) Student Satisfaction and Self-confidence in Learning (SSS): The SSS scale, developed by the National League for Nursing in collaboration with Laredal (30), is completed by students after the course implementation. It consists of two subscales: satisfaction and self-confidence, each comprising 13 items rated on a Likert 5-point scale. Higher scores indicate greater levels of satisfaction and self-confidence.
(3) Educational Practices in Simulation Scale (EPSS): The EPSS measures the extent to which best practice principles are applied in simulation instruction. It consists of four dimensions: self-directed learning, collaboration, learning styles, and high expectations, with a total of 16 items. The scale used is a Likert 5-point scale, and the total score ranges from 16 to 80, with higher scores indicating a higher degree of application of best practice principles in the simulation. The Cronbach’s alpha coefficient of the EPSS is 0.91 (31). The Chinese version of Wang et al. (32) from 2013 was used in this study, with a Cronbach’s alpha coefficient of 0.94.
(4) Jefferson Health Care Cooperation Attitude Scale: This scale, developed by Hojat et al. (33), measures physicians’ and nurses’ attitudes toward healthcare cooperation. The Chinese version by Yang et al. (34) was used in this study. It consists of four dimensions: shared education and teamwork (7 items), nursing vs. treatment (3 items), nurses’ work autonomy (3 items), and physician domination (2 items), with a total of 15 items. The Likert 4-point scale is used, and the total score ranges from 15 to 60, with higher scores indicating a more positive attitude toward healthcare cooperation. A score between 45.01 and 60.00 was considered a high level of healthcare cooperation attitude, while a score between 30.01 and 45.00 was considered moderate, and a score between 15.01 and 30.00 was considered low. Hojat et al. (35) assessed the structural validity, content validity, and reliability of the scale. The Chinese version of the Jefferson Health Care Cooperation Attitude Scale had a Cronbach’s alpha coefficient of 0.848 and a content validity index of 0.893.
(5) Critical Thinking Disposition Inventory-Chinese Version (CTDI-CV): The impact of the curriculum before and after its implementation was assessed using the CTDI-CV, which was translated and revised by Peng et al. (36). The inventory consisted of 70 items, categorized into 7 dimensions: truth-seeking, open-mindedness, analytical ability, systematic ability, self-confidence in critical thinking, intellectual curiosity, and cognitive maturity. Each dimension comprised 10 items. A 6-point scale was used to measure critical thinking ability, ranging from 1 (strongly disagree) to 6 (strongly agree). Some items were reverse scored. The total score ranged from 70 to 420. Scores of 70–210 indicated negative critical thinking ability, 211–279 represented unclear meaning, 280–349 reflected positive critical thinking ability, and 350–420 denoted strong performance. The scale exhibited strong internal consistency, as demonstrated by a Cronbach’s alpha coefficient of 0.90, and content validity with an index of 0.89.
(6) Semi-structured interview: The study conducted one-to-one semi-structured interviews using an interview outline as a basis, as shown in Figure 2. The researcher developed the outline based on a literature review, the study’s purpose, and input from the teaching team. Two students were then selected for pre-interviews to ensure the outline met the research questions’ needs. The final version of the interview outline was formed by the researcher after correcting any misrepresentations of the pre-interviews. The outline included specific elements such as inquiring about the most helpful aspect of the course for personal professional development and identifying strengths and weaknesses in the program’s design and implementation. What suggestions do you have for improving the implementation of the course in the future? The instructor conducted interviews with the students at the end of the course instruction in July 2022. After analyzing the profiles of eight students, no new themes emerged, indicating that data saturation had been reached. The interviews continued with two additional students, resulting in a sample size of ten students.

### 2.3 Statistical analysis

All raw data were entered into an Excel sheet and imported into SPSS 25.0 statistical software for analysis (37). Descriptive statistics, specifically the mean ± standard deviation, were employed to depict the students’ age. Two independent samples *t*-tests were conducted to compare the scores of the attitude toward healthcare cooperation scale and the critical thinking skills scale before and after the course (both scale scores followed a normal distribution). The scores of the simulated teaching practice scale, student learning satisfaction, and self-confidence scales were examined for normality and demonstrated conformity to a normal distribution, thus described using the mean ± standard deviation.

This study employed a phenomenological research methodology (38) to fully comprehend the students’ experience of the course, a widely used approach in the fields of nursing education, nursing administration, and clinical nursing. The collection, transcription, and analysis of interview data were conducted simultaneously. Each respondent’s audio-recorded interview data was transcribed into text within 48 h by a team consisting of Menghan Jiang and Bo Dong. The interview text data were managed, analyzed, and coded using the Colaizzi seven-step analysis method and NVivo 12.0 software (39, 40). Using the above analytical procedures, this study initially labeled the initial data of the ten students as A1–A10 (A1–A3 for clinical medical students, A4–A10 for nursing students). The initial data was then refined and summarized to form sub-themes, denoted as B1–Bn. These sub-themes were further generalized to form the themes of this study, denoted as C1–Cn.

### 2.4 Ethical procedures

The study was approved by the Ethics Committee of Shandong University of Traditional Chinese Medicine before data collection. The researcher provided a comprehensive explanation of the study’s purpose, methods, and significance to the prospective participants, who were given the freedom to decide whether or not to participate after being fully informed. The questionnaire was collected anonymously, and the researcher assured the participants that the personal data collected would be strictly utilized for academic research purposes only. Moreover, the video recordings of the teaching process and the interview content would be treated with utmost confidentiality.

## 3 Results

### 3.1 Student course grades

At the end of the course, the average score of the 22 students ranged from 69.2 to 90.1, with a mean of 79.19 ± 5.12. Out of these, one student scored 90.01 or above, seven students scored between 80.01 and 90, twelve students scored between 70.01 and 80, and two students scored between 60.01 and 70. The scores for each specific subdimension are detailed in Table 5.

**TABLE 5:** Student achievement scores.

	Minimum value	Maximum value	Score (X ± S)
Formative evaluation	38.80	53.30	46.80 ± 3.51
Self-esteem	6.40	9.30	7.87 ± 0.70
Others’ evaluations	13.00	17.60	15.62 ± 1.20
Teacher evaluation	18.00	26.40	23.32 ± 1.87
Summative evaluation	28.80	36.80	32.38 ± 2.01
Totals	69.20	90.10	79.19 ± 5.12

### 3.2 Student satisfaction, self-confidence, and teaching practice scale scores

Table 6 displays the results of the survey on students’ satisfaction with course teaching, self-confidence, and feelings about teaching practice. The mean score for students’ satisfaction with course teaching was 4.44 ± 0.37 (maximum average score of 5), with 21 students (95.45%) scoring 4 or higher, and no students scoring below 3. The mean score for self-confidence was 4.16 ± 0.33 (maximum average score of 5), with 15 students (68%). All students scored 3 or higher, with 18% scoring 4 or higher. Students reported positive perceptions of the teaching practice experience, with all four dimensions of the teaching practice scale receiving high ratings: independent learning, cooperation, multiple learning styles, and high expectations. The dimension with the highest score was multiple learning styles, with a mean score of 4.41 ± 0.40.

**TABLE 6:** Student satisfaction, self-confidence, and teaching practice scale scores.

Scale	Dimensionality	Score (X ± S)
Satisfaction and self-confidence scales	Satisfaction	4.44 ± 0.37
	Self-confidence	4.16 ± 0.33
Simulation of teaching practice scale	Independent learning	4.19 ± 0.27
	Cooperation	4.39 ± 0.34
	Multiple learning styles	4.41 ± 0.40
	High expectations	4.23 ± 0.34

### 3.3 Comparison of students’ attitudes toward healthcare cooperation scores before and after the implementation of the curriculum

Table 7 illustrates the changes in students’ scores on the HealthCare Cooperation Attitude Scale before and after the course. The scores and total scores for the dimensions of shared education and teamwork, job autonomy of nurses, and physicians’ domination were significantly higher after the course, demonstrating statistically significant differences (*P* < 0.01). However, there were no significant differences in the control dimensions of nursing and treatment.

**TABLE 7:** Comparison of students’ attitudes toward healthcare cooperation before and after the implementation of the curriculum (X ± S).

Projects	Pre-teaching	After teaching	*t*	*P*
Shared education and teamwork	22.82 ± 1.99	24.55 ± 1.50	−3.247	0.002***
Comparison of nursing and treatment	9.91 ± 0.87	10.45 ± 1.14	−1.782	0.082
Job autonomy of nurses	9.64 ± 1.36	10.91 ± 0.92	−3.626	0.000***
Physicians’ domination	4.59 ± 1.14	6.68 ± 0.89	−6.768	0.000***
Total score	46.95 ± 2.87	52.59 ± 2.34	−7.135	0.000***

***P < 0.01.

### 3.4 Comparison of student’s critical thinking skills scores before and after the implementation of the curriculum

Table 8 presents the differences in students’ scores and total scores for each dimension of the Critical Thinking Skills Scale before and after the course. Statistically significant differences were observed in the scores and total scores for each dimension, indicating a significant improvement in critical thinking skills after the course. Notably, the comparative differences in scores for the open-mindedness and cognitive maturity dimensions were not statistically significant.

**TABLE 8:** Comparison of students’ critical thinking skills before and after the implementation of the curriculum (X ± S).

Projects	Pre-teaching	After teaching	*t*	*P*
Searching for the truth	34.05 ± 4.99	38.32 ± 4.27	−3.052	0.004***
Open-mindedness	41.86 ± 3.54	42 ± 3.82	−0.123	0.903
Analytical skills	42.41 ± 4.89	45.5 ± 2.86	−2.561	0.014**
Systematic capabilities	38.73 ± 5.16	42.95 ± 2.38	−3.491	0.001***
Self-confidence in critical thinking	39.55 ± 4.81	45.14 ± 4.40	−4.024	0.000***
Desire for knowledge	42.41 ± 3.49	48.14 ± 3.66	−5.318	0.000***
Cognitive maturity	39.27 ± 6.48	41.82 ± 3.70	−1.600	0.117
Total score	278.27 ± 21.85	303.86 ± 13.90	−4.635	0.000***

**P < 0.05,

***P < 0.01.

### 3.5 Results of interviews

This study constructed 11 sub-themes (B1–B11) and 4 themes (C1–C4) by coding, organizing, and analyzing the content of the interviews. C1–stimulating interest in learning and promoting active learning; C2–collaborative learning and improving healthcare collaboration; C3–student-centeredness and promoting the development of clinical thinking skills; and C4–students’ suggestions for curriculum optimization and improvement. The levels and information of specific nodes are shown in Table 9.

**TABLE 9:** Interview results nodes.

Topics	Sub-topic	Example of coding (from the original words of the interviewee)
C1: stimulating interest in learning and promoting active learning	B1: concentration	A2: I can concentrate more than before in class and work with other students to reorganize the theoretical and operational knowledge I had learned and apply it to my training.
	B2: review of knowledge and skills	A7: before the start of each class, we review the theoretical knowledge and operational steps related to the case in advance.
C2: collaborative learning to improve healthcare collaboration	B3: self-perception of role	A8: in this class, I learned what doctors and nurses should do, respectively, in a specific situation in the atmosphere of healthcare collaboration, and had a clearer understanding of the roles they assume.
	B4: leadership	A1: the course is team-based learning, in each class, I can gain, in addition to the case of relevant theories and operational skills more familiar, give me a great feeling is to recognize the power of team leadership, in the face of emergencies, the nurse in charge or attending doctors need to find the condition promptly and report to the superiors, then around the patient-centered team leader needs to be accurate, timely and make the right decision, only then a team can effectively organize and implement the resuscitation.
	B5: medical and nursing communication	A3: I learned some communication strategies in the class, for example, in the class on postpartum hemorrhage in normal labor, I learned how to use the SBAR communication model to report the patient’s condition to the doctor effectively and accurately. I believe that the communication strategies I learned in the class will be very practical in my future clinical work.
	B6: situational awareness	A9: the high-fidelity simulation training session in each class is very tense, I sometimes forget what I am going to do next, but the team members will kindly give me some small reminders so that I can finish the operation smoothly. This shows that when working in a team, we not only need to do our job well but also improve our ability to monitor the situation.
C3: student-centeredness for clinical thinking skills development	B7: identifying and solving problems	A5: the guided feedback was an accomplished session in which I realized that I had many shortcomings, but the teacher and my classmates did not make fun of me, and at the same time, through a few explanations and pointers from the teacher, I was able to know what to do to correct my mistakes.
	B8: adaptability	A10: the complexity of the case scenarios and the progression of the disease in this course gave me a deeper and more systematic understanding of the disease itself as well as the difficulties of clinical work, and greatly enhanced my resilience so that I believe I won’t be alarmed when I encounter situations similar to those in the cases in the future.
	B9: critical thinking	A6: this course has made me bold in expressing my ideas, honed my analytical skills, and improved my logic skills a lot.
C4: student suggestions for course optimization and improvement	B10: increase reflection time	A8: I think the teacher-guided reflection activity can make me better at identifying mistakes, but this session sometimes the teacher imparts a little too much knowledge and speaks a little too fast for me to keep up with the pace, so I hope I can increase the time for reflection and summarization.
	B11: rich case study	A4: I hope the instructor can design more emergencies or rare clinical cases and conduct more of these courses so that we can build a stronger foundation for entering the clinic.

## 4 Discussion

This study developed a simulation training course for medical and nursing collaboration based on the Fink model. The course’s teaching effectiveness was evaluated, and the results showed that all students passed the assessment with a mean grade of 79.19 ± 5.12. The course grades were calculated by combining formative and summative evaluations. Formative evaluations included self-evaluation, peer evaluation, and teacher evaluation. Self-evaluation and peer evaluation promote effective student participation in class. Teacher evaluation, based on group members’ performance, helps teachers focus on individual performance. Video evaluation serves as the summative review for the teacher after teaching the course. This assessment approach is multifaceted, focusing not only on student learning outcomes but also on capturing changes in the learning process.

According to research, best practices in undergraduate education involve seven principles. These include developing reciprocity and cooperation among students, honoring diverse talents and learning styles, and providing timely feedback (41). The Simulated Teaching Practices Scale used in this study can assess the extent to which these principles are implemented. The study results indicate that all dimensions scored above 4, similar to Liu et al’s study (42), suggesting that the course adhered to best practice principles. The course objectives are clearly stated and emphasize independent learning and active participation. This allows for effective communication and idea exchange between students and teachers, with the latter providing guidance to address individual student needs. As a result, students express high satisfaction with the course’s teaching methods, scoring it (4.44 ± 0.37) which is higher than in other studies (43).

Self-confidence is an essential trait for healthcare professionals to possess, as it can greatly impact their clinical decision-making ability and response to emergencies. Research has shown that individuals with higher levels of self-confidence are better equipped to handle the challenges they encounter, particularly in the realm of patient safety (44). Therefore, it is crucial to cultivate self-confidence in medical and nursing students. The study found that the curriculum significantly contributed to the students’ confidence levels, as evidenced by their self-confidence score of (4.16 ± 0.33). This can be attributed to the hands-on opportunities provided by the course, where students were able to apply their knowledge and skills in completing case tasks alongside their team members during high-fidelity simulation training. Such experiences fostered confidence in their abilities and knowledge (45).

According to a study (46), a standardized interprofessional collaborative education program has a positive impact on developing students’ teamwork skills and overall competence. The study found that completing the course significantly improved students’ attitudes toward healthcare cooperation and their scores in three dimensions: shared education and teamwork, job autonomy of nurses, and physicians’ domination (*P* < 0.05). In interviews, students emphasized that the curriculum improved their leadership abilities, communication skills, and ability to work collaboratively. These findings suggest that the curriculum effectively enhanced students’ attitudes toward healthcare cooperation and their collaborative skills, which is consistent with previous research (15, 19). Effective communication and collaboration among healthcare professionals are essential for patient-centered care. However, healthcare professionals may have varying concerns when treating the same patients due to different specialties. Therefore, it is essential to foster teamwork awareness and skills among healthcare professionals. The institutional education stage plays a crucial role in cultivating mutual respect and cooperation among medical students from various disciplines. In this study, students were trained in a high-fidelity simulation through role-playing and group work. This allowed students to understand that nurses are not solely assistants to doctors and that healthcare professionals have equal importance in enhancing patient health outcomes. Additionally, students learned how to follow the process of division of labor among their team members and work collaboratively to complete practical training tasks. This teaching method can enhance students’ attitudes toward healthcare collaboration and help them internalize the concept of interprofessionalism. This, in turn, can lead to effective collaboration in future clinical work (47).

Additionally, the study results revealed no noteworthy distinction in the students’ scores regarding the dimension of “care vs. treatment.” This outcome could be attributed to the students’ regular education in professional knowledge and skills. They already comprehended that healthcare aims to provide quality services to patients. Consequently, they were able to offer physical and mental health education to patients while monitoring the effectiveness of treatment during nursing interventions.

High-fidelity simulation for healthcare collaboration can exercise students’ critical thinking skills. Some studies have measured the level of students’ critical thinking skills by using teachers’ subjective evaluation, which was categorized as excellent, good and fair (48). Whereas many studies assessed students’ critical thinking skills by means of a scale (49, 50), which is more objective. The study utilized the latter approach. The results indicated a positive increase in the total score of students’ critical thinking skills scale after the curriculum was taught (303.86 ± 13.90) compared to before the teaching. Additionally, significant differences (*P* < 0.05) were observed in the scores for the five dimensions of finding the truth, analytical ability, systematic ability, self-confidence in critical thinking, and curiosity. The interview results revealed that students exhibited increased confidence in emergency handling and improvement in clinical thinking skills, such as problem identification and problem-solving, after the implementation of the curriculum.

The enhancement of students’ critical thinking skills in this study can be attributed to the positive learning atmosphere created during the course. Through high-fidelity simulation training sessions, clinical and nursing students collaborated to complete tasks related to teaching cases. This allowed them to effectively provide treatment and care in clinical practice when faced with similar situations, improving their understanding of disease progression and routine management processes. During the review sessions, students had the opportunity to exchange and discuss ideas with teachers and classmates, express their opinions, and exercise their logical thinking and analytical abilities. Self-reflection helped students identify their own shortcomings, motivating them to address gaps in theoretical knowledge and operational skills in a timely manner.

Furthermore, the study revealed no notable distinction in scores regarding the aspects of open-mindedness and cognitive maturity. Two factors may affect students’ perception of simulators vs. real patients: psychological differences and limited opportunities to integrate classroom learning with clinical practice due to lack of hospital internships. To improve integration, students should focus on developing medical and nursing communication skills as well as emergency resuscitation techniques. Their insight and psychological cognition may still be developing, and further observation is needed as they gain more experience.

Although the high-fidelity training course on healthcare cooperation has demonstrated a positive impact on students’ attitudes, abilities in healthcare cooperation, and clinical thinking skills, there are several limitations to consider. Firstly, since this course is the first interprofessional course conducted at our university, there is room for improvement in terms of teaching faculty and their skills. Future efforts should focus on providing further training for faculty in interprofessional education and simulation teaching. Secondly, the sample size was relatively small, and only the initial effects of the course were tested. To objectively analyze the impact of the medical-nursing cooperation training course on students’ performance, future studies should expand the sample size and establish control groups. Furthermore, to enhance the evaluation process, it may be beneficial to include a high-quality scale for assessing students’ medical and nursing cooperation abilities and resilience. Thirdly, a comparative analysis of the attitudes toward healthcare cooperation between clinical medical students and nursing students was not conducted. Further exploration is needed to examine potential differences in attitudes toward healthcare cooperation between these two specialties.

## 5 Conclusion

In this study, we developed a high-fidelity simulation training course on healthcare collaboration based on the Fink model. We implemented the course and verified its teaching effectiveness. The course improved students’ attitudes toward healthcare collaboration and enhanced their critical thinking abilities, promoting cross-fertilization of nursing disciplines and curriculum reform. This provides a reference for the development of healthcare collaboration education.

However, this study still has limitations: Firstly, since this course is the first interprofessional course conducted at our university, there is room for improvement in terms of teaching faculty and their skills. Future efforts should focus on providing further training for faculty in interprofessional education and simulation teaching. Secondly, the sample size was relatively small, and only the initial effects of the course were tested. In the future, as the course progresses, the sample size can be expanded, and control groups can be established to objectively analyze the impact of the medical-nursing collaboration training course on students’ performance. Additionally, incorporating a high-quality scale to assess students’ medical and nursing collaboration ability and resilience would further enhance the evaluation process. Thirdly, a comparative analysis of the attitudes toward healthcare collaboration between clinical medical students and nursing students was not conducted. Further exploration is needed to examine potential differences in attitudes toward healthcare collaboration between these two specialties.

## Data availability statement

The original contributions presented in the study are included in the article/supplementary material, further inquiries can be directed to the corresponding author.

## Ethics statement

The studies involving humans were approved by the Medical Ethics Committee of School of Nursing, Shandong University of Traditional Chinese Medicine. The studies were conducted in accordance with the local legislation and institutional requirements. The participants provided their written informed consent to participate in this study.

## Author contributions

M-HJ: Conceptualization, Methodology, Validation, Writing – original draft, Writing – review and editing. L-WD: Data curation, Formal Analysis, Writing – review and editing. BD: Data curation, Formal Analysis, Writing – original draft, Writing – review and editing. MZ: Methodology, Writing – original draft. Y-PL: Data curation, Writing – original draft. C-XL: Funding acquisition, Writing – review and editing.
